# Physical fitness and health-related quality of life in nursing students: a cross-sectional study with a gender perspective

**DOI:** 10.1186/s12889-026-27062-4

**Published:** 2026-03-21

**Authors:** Alberto Bermejo-Cantarero, Victoria Mazoteras-Pardo, Ana María Recio-Vivas, Laura Muñoz de Morales-Romero, Raúl Expósito-González, Sandra Martínez-Rodríguez, José Miguel Mansilla-Domínguez

**Affiliations:** 1 Faculty of Nursing of Ciudad Real, Universidad de Castilla-La Mancha, Ciudad Real, Spain; 2 Centro de Estudios Sociosanitarios (CESS), Universidad de Castilla-La Mancha, Cuenca, Spain; 3 Faculty of Medicine, Health and Sports, Department of Nursing, Universidad Europea de Madrid, Villaviciosa de Odón, Madrid, Spain

**Keywords:** Physical fitness, Health-related quality of life, young adult, University students, Gender differences

## Abstract

**Background:**

Health-related quality of life (HRQoL) is a key indicator of well-being among university students, who are often exposed to stress and unhealthy lifestyles. Students enrolled in nursing programs represent a specific subgroup within the university population, characterized by high academic demands and early exposure to health-related knowledge and practices. Although higher physical fitness (PF) levels have been associated with better HRQoL, the contribution of specific PF components and potential gender differences has been insufficiently explored. This study aimed to examine the association between PF and HRQoL in nursing students, with a focus on gender differences and the role of individual PF components across HRQoL dimensions.

**Methods:**

A cross-sectional analysis of 637 nursing students from two Spanish universities was conducted. HRQoL was assessed using the WHOQOL-BREF questionnaire. Self-reported PF was assessed using the International Fitness Scale (IFIS) questionnaire. Body mass index and parental education level were included as covariates. Partial correlations, analysis of covariance (ANCOVA), and linear regression analyses were performed.

**Results:**

Higher PF levels were associated with better HRQoL across all dimensions. General PF, cardiorespiratory fitness (CRF), muscular strength (MS), and speed-agility showed the strongest associations with HRQoL. Men reported higher PF levels, greater psychological well-being, and higher overall QoL compared to women. Flexibility showed statistically significant associations with physical and psychological well-being in women, and weaker associations with life satisfaction and overall QoL in the total sample. Stronger associations between PF and HRQoL were observed for general PF, CRF, and MS, especially in analyses conducted among men.

**Conclusions:**

Higher levels of general PF and its core components are closely associated with most dimensions of HRQoL among nursing students. From a gender perspective, the observed sex-related patterns suggest the potential value of gender-sensitive interventions to improve HRQoL through PF within this population.

**Supplementary Information:**

The online version contains supplementary material available at 10.1186/s12889-026-27062-4.

## Background

Health-related quality of life (HRQoL) encompasses individuals’ experiences, beliefs, and perceptions concerning the physical, psychological, and social dimensions of health and functioning. It refers to a person’s self-perceived health status in terms of well-being and functional capacity across various domains of daily life [1]. Over the past decades, considerable attention has been devoted to this construct, as it is recognized as a key global health outcome [2].

Among young adults in university settings, HRQoL is often negatively influenced by the high prevalence of stress, depression, and anxiety symptoms within this population [3]. Moreover, the transition to university life frequently entails substantial lifestyle changes, increasing the risk of adopting unhealthy habits that may adversely affect HRQoL [4, 5].

Physical fitness (PF), defined as the ability to perform physical activity with vigor and efficiency, is considered a key determinant of health and well-being [6]. The health-related components of PF are cardiorespiratory fitness (CRF), muscular endurance, muscular strength (MS), body composition, and flexibility. In addition, skill-related components such as speed–agility (S-A) reflect neuromuscular coordination, movement efficiency, and the ability to perform rapid and complex motor tasks [7]. The decline in physical activity levels commonly observed during the university years [8, 9] may lead to a reduction in PF. Moreover, health-related PF can be influenced by additional factors such as body weight and socioeconomic status [10, 11]. Previous studies have shown that higher levels of PF are associated with improved academic and cognitive performance [12], as well as lower levels of anxiety and depression [13, 14], thereby enhancing perceived physical and psychological health [15]. Moreover, adults who engage in aerobic and muscle-strengthening activities at recommended levels exhibit a reduced risk of all-cause mortality, as well as mortality from specific causes such as cardiovascular diseases, cancer, and chronic respiratory conditions [16–18].

Studies examining the relationship between different components of PF and HRQoL in young adults are scarce, particularly those simultaneously considering multiple fitness components beyond CRF and MS, and their differential associations with specific HRQoL domains by sex. Although some studies have shown that higher levels of PF are associated with better HRQoL in young men [19–21], in many cases not all components of PF and their relationship with different dimensions of HRQoL have been analyzed. Furthermore, an important limitation of these studies is the lack of analysis of gender differences, despite the fact that, at this age, women tend to be less physically active and have poorer PF and HRQoL than men [22–24].

Given that this stage of life is critical for the establishment of long-term health habits, and considering the high prevalence of mental health problems among university students [25], understanding the relationship between PF and HRQoL in young adults within the university setting could provide valuable insights for designing more effective health promotion strategies targeting this population group.

From a theoretical perspective, the relationship between PF and HRQoL can be conceptualized within a biopsychosocial framework [1, 26]. Higher levels of PF may influence HRQoL through interconnected physiological mechanisms, such as improved inflammatory profiles, enhanced neuroendocrine stress regulation, and greater neuromuscular efficiency [27–29]; psychological mechanisms, including increased self-efficacy, improved body image, and more effective emotional regulation [30–32] and behavioral or social mechanisms, such as better sleep quality, greater functional autonomy, and increased opportunities for social interaction [33–35]. Importantly, different components of PF may contribute differentially to specific HRQoL domains, and these pathways may operate differently according to gender [6, 21, 22]. Based on this framework, the present study examines not only the association between overall PF and HRQoL, but also the role of individual fitness components and potential gender-specific patterns across HRQoL dimensions. Therefore, the present study aims to analyze the association between PF and HRQoL in young adult nursing students within the university setting, focusing on gender differences and the impact of different components of PF on each dimension of HRQoL.

## Materials and methods

### Study design and population

This is a cross-sectional study in which 700 students were invited to participate, of whom 637 (91%) accepted. The participants were from two universities (one public and one private) located in the autonomous communities of Castilla-La Mancha and Madrid, Spain. All participants were enrolled in the nursing degree program during the data collection period (academic year 2024/2025). The percentage of women enrolled in health sciences programs at these universities is higher than the percentage of men and is similar to the gender distribution of the participants in this study (i.e., ~ 75% women). An a priori power analysis was conducted using G*Power version 3.1.9.7 to determine the minimum sample size required to test the study hypothesis. The results indicated that the required sample size to achieve 95% power for detecting a medium effect (η² = 0.06), with a significance criterion of α = 0.05, was *N* = 400 for an ANCOVA statistical test. Simultaneously, the results indicated that the sample size required to achieve a power (1-β) = 0.95, with a significance criterion of α = 0.05 and a correlation p H1 = 0.3, was *N* = 138 for the normal bivariate correlation statistical test.

### Ethical and legal aspects

The study protocol was approved by the Research Committee of the Research Commission of the Doctoral and Research School of the European University of Madrid, Spain (Code: 2024 − 789). Additionally, this study complied with Spain’s Organic Law on Data Protection [36] and the Declaration of Helsinki [37].

### Data collection

After obtaining the approval of the heads of each faculty, an informational meeting was held with the student body to explain the objectives and ethical issues of the project. The professors and researchers of this study were responsible for providing a Google Forms link with the questionnaires. These were completed in person during class time to minimize the loss of potential participants.

### Measures and Instruments

Sociodemographic data were collected by questionnaire, including gender, age, mother’s education level, father’s education level, weight, and height (body mass index (BMI) was calculated from these measurements). Parental education level was operationalized as the highest educational attainment achieved by either parent and was included as an indicator of family socioeconomic background. BMI was categorized as underweight (< 18.5), normal weight (18.5–24.9), overweight (25.0-19.9), and obese (≥30), according to the World Health Organization (WHO) cut-off points for adults [38].

### Health-related quality of life

The Spanish-adapted version of the generic WHOQoL-BREF questionnaire [39] was used to assess participants’ HRQoL. In the context of the present study, HRQoL was operationalized as individuals’ perceived physical, psychological, social, and environmental functioning in relation to health and daily life, consistent with a biopsychosocial public health perspective. This questionnaire consists of 26 items; each rated on a 5-point Likert scale [1–5]. The first two items assess overall perception of quality of life (QoL) and satisfaction with health, respectively. The remaining 24 items are distributed across four domains: (I) Physical well-being (seven items), which covers aspects such as pain/discomfort, energy or fatigue levels, sleep and rest quality, need for medication, mobility, performance of daily activities, and capacity for work.; (II) Psychological well-being (six items), which assesses positive and negative feelings, self-esteem, ability to think, learn, remember, and concentrate, body image perceptions, and spirituality, religion, or personal beliefs; (III) Social relationships (three items), which include interpersonal relationships, sexual activity, and practical social support; and (IV) Environment (eight items), which considers factors such as financial resources, access to information and skill development, leisure and recreation, home environment, availability of health and social care, physical safety, environmental quality, and transportation. Although the WHOQOL-BREF was originally developed as a generic QoL instrument, it has been widely used in public health research to assess HRQoL by capturing multidimensional aspects of functioning and well-being closely linked to health [40, 41]. The mean score of each domain is used to calculate a raw score for that domain. Following WHO guidelines, the sum of the raw scores can be converted to a 0–100 scale, where higher scores indicate better HRQoL. The WHOQoL-BREF has previously been used to assess HRQoL among health sciences university students [42–44], and the Spanish version has shown positive psychometric properties [45].

### Self-reported fitness

Self-reported PF was assessed using the International Fitness Scale (IFIS) [46]. The IFIS consists of five Likert-scale questions regarding participants’ self-perceived overall PF, CRF, S-A, and flexibility. For each item, the response options range from “very poor” to “very good.” Higher scores indicate better PF. This questionnaire has been previously validated in young Spanish university adults [47], and has been shown to be a good alternative to field test measures of PF in large sample studies.

### Statistical analysis

The fit of each variable to a normal distribution was assessed using both graphical methods and the Kolmogorov-Smirnov test. Descriptive statistics (mean and standard deviation for quantitative variables and frequencies and percentages for categorical variables) were used to describe the baseline characteristics of the participants. The independent samples t-test was used to assess differences between continuous variables, while the chi-squared test was used for descriptive analyses of categorical variables. Cronbach’s alpha was used to assess the reliability of the scales, with values of 0.75 for the IFIS scale and 0.86 for the WHOQoL-BREF scale.

Partial correlation coefficients were calculated to examine the relationship between the HRQoL dimensions and the different components of PF, by sex. A value < 0.1 was considered trivial, 0.1–0.29 small, 0.3–0.49 moderate, and values ≥ 0.5 large.

Analysis of covariance (ANCOVA), with Bonferroni-adjusted post hoc tests applied to all pairwise comparisons between PF categories within each model, was used to assess the differences in mean scores of the WHOQoL-BREF dimensions across the categories of the PF components included in the IFIS. Partial eta squared (η²_p_) was calculated for the ANCOVA test, indicating small effect sizes (0.01–0.06), medium (≥ 0.06–0.14), and large (≥ 0.14) [48].

In addition, several linear regression analyses were conducted, using the WHOQoL-BREF dimensions as dependent variables. General PF, CRF, MS, S-A, and flexibility were included as independent variables. The assumptions of linearity were checked, and multicollinearity was assessed using the variance inflation factor (VIF). VIF values were below 5 in all analyses, indicating no multicollinearity. All analyses were conducted controlling for BMI and parental education level by sex, as these variables are known to influence both PF and HRQoL through biological and socioeconomic pathways. Age was not included as a covariate due to the relatively narrow age range of the sample and its limited variability within this population of nursing students. Analyses were conducted using complete-case analysis. The proportion of missing data was low across variables, and no imputation methods were applied. Data were analyzed using IBM SPSS Statistics version 25 with a significance level set at *p* < 0.05. The heatmap was created using Python (version 3.13.2) and the Seaborn library (version 0.13.2), applying a diverging color scale (“coolwarm”) to reflect positive and negative associations. All values were displayed within the cells, and the color gradient was scaled consistently across the matrix for interpretability.

## Results

Table 1 presents the main descriptive characteristics of the sample by sex. No differences were observed in age, BMI, or parents’ educational level according to sex. A total of 17.9% of participants were overweight (26% of men and 15.8% of women), and 4.9% were classified as obese (5.2% of women and 4.7% of men). Compared to women, men scored higher in all components of PF, except for flexibility, where no significant differences were observed (*p* = 0.114). The mean scores for each of the WHOQoL-BREF dimensions were similar between women and men, except for psychological well-being (*p* = 0.003) and overall QoL perception (*p* = 0.024), both of which were higher in men.

**Table 1:** Characteristics of the sample by gender

		Total		Women		Men		
		*n*	% or M(SD)	*n*	% or M(SD)	*n*	% or M(SD)	*p*
Age	596		22.57 (5.92)	455	22.46 (5.75)	141	23.01 (6.55)	0.376
BMI	625		23.09 (4.50)	475	22.97 (4.82)	150	23.46 (3.27)	0.243
Underweight	42		6.6	34	7.2	8	5.3	
Normal weight	434		68.1	339	71.4	95	63.3	
Overweight	114		17.9	75	15.8	39	26.0	
Obesity	31		4.9	24	5,1	7	4.7	
Mother’s educational level	637			487		150		0.914
Compulsory basic education	215		33.80	168	35.30	47	31.1	
Secondary Education	146		22.90	109	22.90	37	24.7	
University	219		34.40	163	34.30	56	37.3	
Masters or Doctorate	57		8.90	47	9.70	10	6.7	
Father’s educational level	637			487		150		0.194
Compulsory basic education	267		41.90	212	44.65	55	36.7	
Secondary Education	147		23.10	113	23.80	34	22.7	
University	159		25.00	112	23.53	47	31.3	
Masters or Doctorate	58		9.10	46	9.71	12	8.0	
General PF	636		3.46 (0.82)	487	3.40 (0.81)	149	3.59 (0.86)	**0.020**
CRF	636		3.14 (0.95)	487	3.08 (0.95	149	3.35 (0.91)	**0.003**
MS	636		3.36 (0.91)	487	3.26 (0.90)	149	3.71 (0.84)	**< 0.001**
S-A	636		3.39 (0.87)	487	3.32 (0.86)	149	3.61 (0.86)	**< 0.001**
Flexibility	636		3.14 (1.08)	487	3.18 (1.05)	149	3.01 (1.15)	0.114
HRQoL								
Overall QoL	632		67.01 (18.76)	472	66.05 (18.30)	148	69.76 (20.05)	**0.024**
Life satisfaction	633		67.58 (21.24)	485	67.39 (20.97)	148	67.73 (22.45)	0.600
Physical well-being	628		70.98 (14.03)	469	70.36 (14.22)	148	72.70 (13.29)	0.069
Psychological well-being	630		63.38 (16.26)	470	62.43 (15.99)	148	66.68 (16.44)	**0.003**
Social	632		70.30 (18.29)	468	69.84 (18.68)	149	71.34 (17.35)	0.424
Environment	630		69.04 (13.78)	471	68.51 (13.78)	148	70.45 (13.46)	0.078

Data are presented as means and standard deviation, except for BMI categories and parents’ level of education, which are presented as frequencies and percentages

Differences between sexes were tested with Chi-square and T-test for independent samples

In bold, differences between women and men

*BMI* Body mass index, *PF* Physical Fitness, *CRF* Cardiorespiratory Fitness, *MS* Muscular Strength, *S-A* Speed-Agility

Partial correlation coefficients between the HRQoL domains and general PF, CRF, MS, S-A, and flexibility, for the total sample and by sex, controlling for BMI and parents’ educational level, are presented in Table 2. The independent variables were related to each other, with a correlation coefficient between moderate and large, except for flexibility, which was related only to the rest of the PF components in women, and to S-A in men. Overall, general PF, MS, and S-A were positively associated with all HRQoL domains, except for social relationships in men. CRF was also associated with all HRQoL domains, except for social relationships, in both men and women. Flexibility was associated with overall QoL (*r* = 0.207, *p* < 0.001), life satisfaction (*r* = 0.105, *p* < 0.001), and physical well-being (*r* = 0.124, *p* < 0.001), but only in women.

**Table 2:** Partial correlation coefficients (r) between physical fitness components, and health-related quality of life domains by sex, controlling by body mass index and parents’ educational level

		General PF	CRF	MS	V-A	Flex	Overall QoL	LS	PWB	PsWB	Social
CRF	Total	0.602**									
	Women	0.613**									
	Men	0.567**									
MS	Total	0.541**	0.451**								
	Women	0.506**	0.472**								
	Men	0.643**	0.315**								
V-A	Total	0.534**	0.523**	0.442**							
	Women	0.502**	0.528**	0.428**							
	Men	0.583**	0.464**	0.407**							
Flex	Total	0.176**	0.176**	0.183**	0.295**						
	Women	0.214**	0.228**	0.239**	0.318**						
	Men	0.077	0.066	0.090	0.280**						
Overall QoL	Total	0.393**	0.319**	0.292**	0.290**	0.179**					
	Women	0.367**	0.294**	0.283**	0.282**	0.207**					
	Men	0.438**	0.369**	0.291**	0.271**	0.121					
LS	Total	0.280**	0.216**	0.203**	0.189**	0.087*	0.623**				
	Women	0.254**	0.182**	0.188**	0.164**	0.105**	0.616**				
	Men	0.343**	0.321**	0.255**	0.238**	0.063	0.642**				
PWB	Total	0.277**	0.180**	0.212**	0.258**	0.078	0.402**	0.460**			
	Women	0.239**	0.154**	0.161**	0.238**	0.124**	0.410**	0.457**			
	Men	0.350**	0.232**	0.325**	0.261**	-0.052	0.329**	0.449**			
PsWB	Total	0.330**	0.232**	0.258**	0.265**	0.052	0.428**	0.671**	0.563**		
	Women	0.301**	0.202**	0.210**	0.246**	0.107*	0.424**	0.671**	0.561**		
	Men	0.384**	0.288**	0.297**	0.274**	-0.064	0.397**	0.669**	0.515**		
Social	Total	0.174**	0.055	0.134**	0.151**	0.039	0.322**	0.460**	0.366**	0.520**	
	Women	0.189**	0.061	0.141**	0.159**	0.053	0.347**	0.506**	0.385	0.553**	
	Men	0.114	0.010	0.089	0.111	0.032	0.223**	0.295**	0.262**	0.417**	
Environment	Total	0.263**	0.190**	0.217**	0.263**	0.049	0.440**	0.514**	0.612**	0.584**	0.463**
	Women	0.232	0.174**	0.168**	0.259**	0.054	0.439**	0.522	0.617**	0.597**	0.460**
	Men	0.312**	0.221**	0.315**	0.197*	0.059	0.392**	0.462**	0.556**	0.505**	0.467**

*PF *Physical fitness,* CRF *Cardiorespiratory fitness, *MS *Muscular strength, *V-A *Velocity-Agility, *Flex *Flexibility, *QoL *Quality of Life, *LS *Life satisfaction, *PWB *Physical Well-being, *PsWB *Psychological well-being

In bold, statistically significant relationships

**The correlation is significant at the 0.01 level (bilateral), *The correlation is significant at the 0.05 level (bilateral)

Mean differences in HRQoL dimensions by PF categories, controlling for BMI and parents’ educational level, are included as Supplementary Tables 1 and in Table 3, which presents the results of the ANCOVA analysis. Overall, HRQoL was better among participants with higher levels of PF, in both men and women. An improvement in PF was significantly associated with higher scores across all HRQoL dimensions. These scores increased as PF improved, with the greatest increases observed at good and very good PF levels, and minimal differences between poor and very poor levels. These relationships were consistent across all HRQoL dimensions and all components of PF, except for flexibility, which was only associated with overall QoL in the total sample (η²*p* = 0.073, *p* = 0.001) and in women (η²*p* = 0.072, *p* < 0.001). On the other hand, although there was a trend toward higher mean scores in physical well-being among men with better CRF, the relationship was not statistically significant. Similarly, no statistically significant associations were observed between PF components and the social dimension in men. The largest effect sizes were observed for the relationships between general PF and psychological well-being (η²*p* = 0.148, *p* < 0.001), life satisfaction (η²*p* = 0.156, *p* < 0.001), and overall QoL (η²*p* = 0.227, *p* < 0.001) in men. Similarly, CRF (η²*p* = 0.207, *p* < 0.001) and MS (η²*p* = 0.181, *p* < 0.001) were strongly associated with overall QoL in men. Among women, the largest effect sizes were found for the association between MS and physical well-being (η²*p* = 0.340, *p* = 0.004), and between general PF and overall QoL (η²*p* = 0.122, *p* < 0.001).

**Table 3:** Analysis of covariance (ANCOVA) testing mean differences in health-related quality of life scores by physical fitness categories, controlling for body mass index and parents’ educational level, by gender

		General PF			CRF			MS			V-A			Flex		
		F	*p*	η²*p*	F	*p*	η²*p*	F	*p*	η²*p*	F	*p*	η²*p*	F	*p*	η²*p*
**Overall QoL**	Total	26.410	**0.000**	0.154	16.829	**0.000**	0.114	16.277	**0.000**	0.095	10.650	**0.000**	0.073	4.943	**0.001**	0.031
	Women	16.041	**< 0.001**	0.122	11.885	**< 0.001**	0.094	9.167	**< 0.001**	0.074	8.858	**< 0.001**	0.072	5.021	**< 0.001**	0.042
	Men	10.074	**< 0.001**	0.227	8.938	**< 0.001**	0.207	10.172	**< 0.001**	0.181	1.365	0.249	0.038	2.295	0.062	0.063
**LS**	Total	13.527	**< 0.001**	0.087	7.875	**< 0.001**	0.055	7.875	**< 0.001**	0.048	3.805	**0.005**	0.030	1.282	0.276	0.010
	Women	8.060	**< 0.001**	0.065	4.617	**0.001**	0.039	4.617	**0.004**	0.033	2.463	**0.044**	0.021	1.807	0.126	0.016
	Men	6.337	**< 0.001**	0.156	4.521	**0.002**	0.117	4.521	**< 0.001**	0.132	2.898	**0.024**	0.078	1.160	0.331	0.033
**PWB**	Total	10.612	**0.000**	0.072	5.405	**0.000**	0.036	8.309	**0.000**	0.056	8.440	**0.000**	0.058	1.218	0.302	0.009
	Women	7.186	**< 0.001**	0.059	3.347	**0.010**	0.028	3.970	**0.004**	0.340	5.967	**< 0.001**	0.050	1.878	0.113	0.016
	Men	4.729	**0.001**	0.121	1.740	0.145	0.048	6.911	**< 0.001**	0.131	5.975	**< 0.001**	0.149	0.644	0.632	0.018
**PsWB**	Total	18.238	**0.000**	0.107	10.559	**0.000**	0.063	11.111	**0.000**	0.065	10.173	**0.000**	0.059	0.368	0.832	0.002
	Women	10.821	**< 0.001**	0.086	6.195	**< 0.001**	0.051	5.741	**< 0.001**	0.048	6.163	**< 0.001**	0.051	1.467	0.211	0.013
	Men	5.968	**< 0.001**	0.148	3.442	**0.010**	0.091	4.471	**0.005**	0.089	2.990	0.330	0.061	1.365	0.249	0.038
**Social**	Total	5.363	**0.000**	0.039	2.533	**0.039**	0.018	2.923	**0.021**	0.022	3.786	**0.005**	0.023	1.079	0.366	0.008
	Women	5.555	**< 0.001**	0.046	2.778	**0.027**	0.024	2.898	**0.022**	0.025	3.391	**0.009**	0.029	0.858	0.489	0.007
	Men	0.871	0.483	0.025	0.823	0.512	0.023	0.746	0.527	0.016	0.764	0.551	0.022	0.848	0.497	0.024
**Environment**	Total	9.625	**0.000**	0.061	5.744	**0.000**	0.038	7.640	**0.000**	0.048	8.412	**0.000**	0.058	1.806	0.126	0.012
	Women	5.135	**< 0.001**	0.043	3.972	**0.004**	0.033	3.563	**0.007**	0.030	7.732	**< 0.001**	0.063	1.227	0.298	0.011
	Men	5.096	**< 0.001**	0.130	4.172	**0.003**	0.109	5.552	**0.001**	0.107	1.258	0.290	0.035	0.984	0.419	0.028

In bold, statistically significant relationships

*PF* physical fitness, *CRF* Cardiorespiratory fitness, *MS* Muscular strength, *V-A* Velocity-Agility, *Flex* Flexibility, *QoL* Quality of Life, *LS* Life satisfaction, *PWB* Physical Well-being, *PsWB* Psychological well-being

**Fig. 1:**
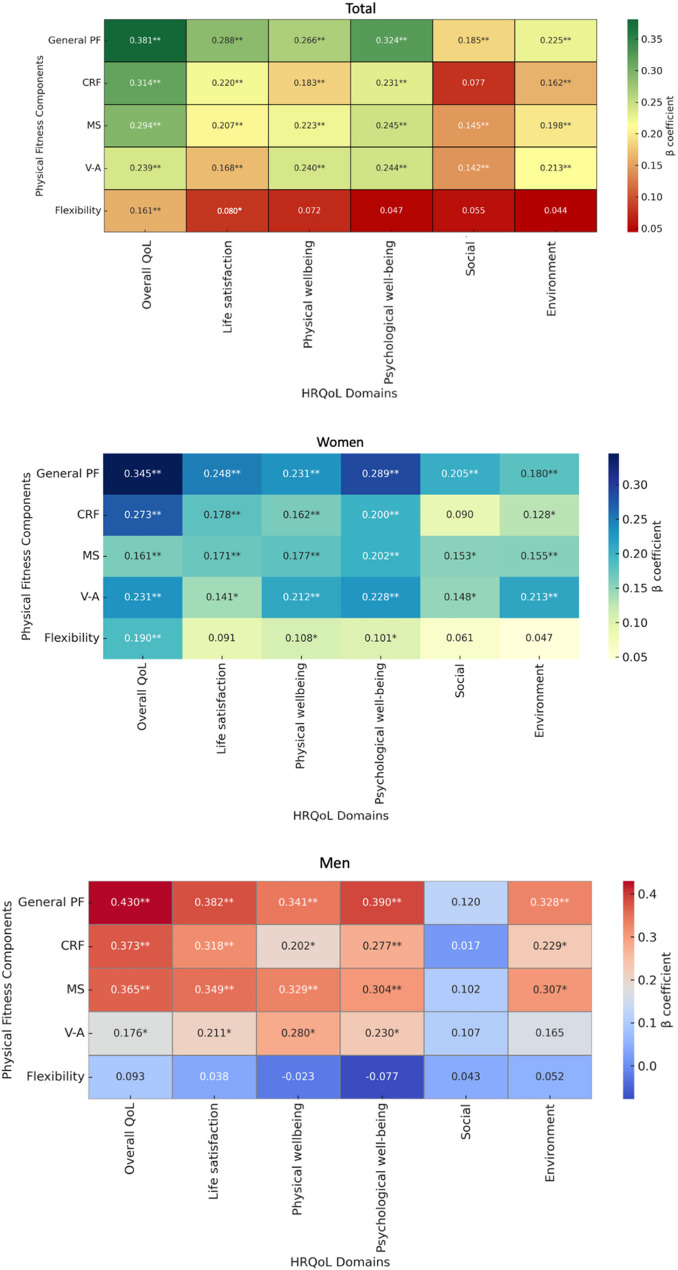
Heatmaps showing the standardized β coefficients representing the independent associations between physical fitness components (General physical fitness, cardiorespiratory fitness, muscular strength, speed-agility, and flexibility) and HRQoL domains, presented for the total sample and by sex. CRF, cardiorespiratory fitness; MS, muscular strength; S-A, speed-agility; HRQoL, health-related quality of life. Associations were derived from multiple linear regression analyses, adjusted for body mass index and parental education level. Separate heatmaps are shown for the total sample, women, and men. An asterisk (*) indicates *p* < 0.05, and a double asterisk (**) indicates *p* < 0.001

The independent association between the IFIS components and HRQoL, controlling for BMI and parents’ educational level by gender, is presented in Fig. 1 and in Supplementary Table **2**. In both women and men, physical well-being, psychological well-being, life satisfaction, and overall QoL were associated with general PF, CRF, MS, and S-A. The environmental domain was associated with general PF, CRF, and MS in both women and men, and with S-A only in women (β = 0.148, *p* = 0.002). A statistically significant association was observed between flexibility and physical well-being (β = 0.108, *p* = 0.020) and psychological well-being (β = 0.101, *p* = 0.030) in women, as well as with life satisfaction (β = 0.080, *p* = 0.047) and overall QoL (β = 0.161, *p* < 0.001) in the total sample.

## Discussion

The results of the present study indicated that better self-reported PF was associated with higher HRQoL among nursing students, with some gender-specific differences. Overall, men reported better PF as well as greater psychological well-being and overall QoL compared to women. General PF emerged as the component most strongly associated with HRQoL and was shown to be an important predictor of physical well-being, psychological well-being, environment, life satisfaction, and overall QoL perception in both sexes. MS and V-A also showed a strong association with overall QoL perception in men. In contrast, flexibility showed statistically significant associations with physical and psychological well-being in women, and to life satisfaction and overall QoL in the total sample. These findings indicate that flexibility has a weaker and less consistent relationship with HRQoL—particularly among men—compared with other PF components. In addition, the environmental dimension of HRQoL was associated with overall PF, CRF, and MS in men and women, and with V-A only in women, suggesting that different components of PF may be differentially associated with perceived HRQoL according to gender. In this context, the present findings extend existing evidence by highlighting that not all PF components contribute equally to HRQoL, and that their relevance may differ according to both HRQoL domain and sex. These sex-related differences are based on stratified analyses and therefore reflect observed patterns rather than statistically tested interaction effects.

Interpreted within a biopsychosocial framework, the associations observed between PF and HRQoL likely reflect the convergence of multiple interrelated mechanisms. From a physiological perspective, higher levels of CRF and MS have been associated with improved metabolic efficiency, reduced low-grade inflammation, and more adaptive neuroendocrine responses to stress, which may directly enhance both physical and psychological well-being [27–29]. From a psychological perspective, greater PF is consistently linked to higher self-efficacy, perceived competence, and more positive body image, all of which are key determinants of psychological HRQoL and life satisfaction [30–32, 49]. Behavioral mechanisms may also contribute, as individuals with better fitness often report improved sleep quality, higher energy levels, and greater capacity to engage in academic, recreational, and social activities [33–35]. Importantly, different components of PF may contribute differentially to specific HRQoL domains, and these pathways may operate differently according to gender, as suggested by the distinct association observed between flexibility and psychological well-being in women in the present study. In this context, the stronger associations observed for general PF, CRF, and MS with physical and psychological HRQoL domains may reflect their closer links to metabolic efficiency, inflammatory regulation, and stress-related neuroendocrine pathways, which are central to perceptions of energy, vitality, and emotional well-being. By contrast, the more specific associations observed for flexibility—particularly with psychological well-being and life satisfaction in women—may be better explained by psychosocial mechanisms such as body awareness, emotional regulation, and positive body perception, rather than by direct physiological pathways. These differential patterns support the notion that distinct components of PF may influence specific HRQoL dimensions through partially overlapping but component-specific mechanisms. It should be noted that PF was assessed as a self-perceived construct, which may be influenced by subjective factors such as mood, body image, or social comparison; therefore, the associations observed reflect perceived rather than objectively measured fitness.

In line with this conceptual framework, our findings are consistent with previous studies reporting positive associations between PF and HRQoL across the lifespan [6, 50] and among individuals with various health conditions [51, 52]. Previous research in university populations has similarly shown that regular participation in fitness-related activities is associated with higher levels of physical, mental, and emotional dimensions of HRQoL [53].

When examining individual components of PF, differential patterns of association with HRQoL emerged, and these associations appeared to vary according to sex. A previous study conducted in young men [21] reported that moderate levels of PF were associated with better HRQoL in the physical domain, whereas benefits in the mental domain were only evident among those who achieved the highest level of fitness. Moreover, MS did not show an independent association with HRQoL. In contrast, our results suggest a progressive improvement in both physical and psychological HRQoL domains as PF components increase, with CRF, MS, and V–A each showing independent associations, consistent with a graded association across increasing levels of PF [18]. These findings support the idea that a balanced development across different components of PF may contribute to a broader sense of well-being and life satisfaction.

Our findings are consistent with previous studies showing that men tend to have higher levels of PF [22, 54, 55] and report better perceived HRQoL than women [5, 56]. These differences may be partly explained by sex-related variations in motivational factors associated with physical activity [57] and underlying psychological mechanisms. Intrinsic motivation—based on enjoyment, mastery, or challenge—tends to be more common in men and is associated with sustained engagement in physical activity and better physical outcomes. In contrast, extrinsic motivation—related to weight control or appearance—more frequently reported by women, may lead to less consistent engagement and lower psychological benefits, particularly when linked to body image dissatisfaction [58, 59]. Additionally, lower HRQoL levels among women may be influenced by greater perceived academic stress [60, 61], a higher prevalence of mental health problems [62–64] and a more negative overall life perception [65].

In addition to the physical and mental components, social functioning has previously been associated with better PF [66]. However, in our sample, associations between PF and social and environmental aspects of HRQoL appeared less consistent and of smaller magnitude than those observed for physical and psychological domains, particularly among men. This suggests that improvements in PF do not necessarily translate into enhanced social functioning. One possible explanation is that the social benefits of physical activity may depend more on contextual factors—such as participation in group-based exercise or team sports—than on fitness level itself [35, 67]. Students who engage primarily in individual activities (e.g., gym workouts or running) may experience fewer opportunities for social interaction, limiting the perceived impact of PF on social well-being.

Whereas previous research on PF and HRQoL has primarily focused on CRF and MF [20, 21, 68], in our study, regression analysis showed that after adjusting for BMI and parental educational level, flexibility was associated with better life satisfaction and overall QoL. However, these associations were of small magnitude, and their practical relevance should therefore be interpreted with caution. Among women, flexibility was also associated with greater physical and psychological well-being. These findings highlight the importance of flexibility as a relevant but often overlooked component of PF that may contribute to perceived well-being, particularly in women. Health promotion programs in university settings should therefore consider incorporating flexibility-oriented activities to enhance both physical and psychological aspects of HRQoL.

### Limitations

Several limitations of this study should be considered. First, its cross-sectional nature does not allow for causal inferences. Although higher levels of PF may contribute to better HRQoL, it is also plausible that individuals with more favorable HRQoL are more motivated and able to engage in regular physical activity, thereby improving their PF. Consequently, the observed associations should be interpreted as correlational rather than causal. Secondly, the data were obtained through self-report, which implies the possibility that some participants may have provided inaccurate responses, either intentionally or unintentionally, to some of the questions. In this regard, PF was assessed as self-perceived PF using the IFIS, which captures subjective perceptions rather than objectively measured fitness; such perceptions may be influenced by psychosocial factors (e.g., mood, body image, or social comparison). As a self-report measure, it may be susceptible to common method bias and shared variance with self-reported HRQoL outcomes. Nevertheless, the use of valid and reliable instruments [46, 47] and the assurance of anonymity likely helped minimize such bias. Third, several relevant contextual and personal factors—such as students’ living arrangements (with parents or independently), economic income, and the presence of chronic health conditions—were not included in this study. In addition, other potentially relevant confounders, such as physical activity patterns, dietary behaviors, stress levels, or mental health status, were not assessed, which may have resulted in residual confounding of the observed associations. These factors may significantly influence both PF and HRQoL. Including these variables in future research could therefore provide a more comprehensive understanding of how environmental and health-related factors interact with PF to shape HRQoL outcomes. Fourth, regression results were reported without 95% confidence intervals, which may limit the precision of effect size interpretation and the assessment of estimate uncertainty. Fifth, the relatively large number of statistical tests performed may increase the risk of Type I error. Although Bonferroni corrections were applied to post-hoc analyses, no global adjustment for multiple comparisons was implemented; therefore, findings with small effect sizes or borderline statistical significance should be interpreted with caution. Finally, the sample was composed exclusively of nursing students, with a high proportion of women, which may have affected the results, as nursing students typically possess higher health literacy, greater awareness of healthy behaviors, and stronger self-perceptions of health than peers from other academic disciplines [69, 70]. This sample composition may limit the generalizability of the observed effect sizes and sex-specific patterns, and the smaller male subsample may have reduced statistical power to detect weaker associations in sex-stratified analyses. Consequently, the findings may not be generalizable to the broader university population. Future studies including students from diverse educational backgrounds are needed to confirm whether these associations hold across different fields of study and cultural contexts. Overall, these limitations suggest that while the observed relationships between PF and HRQoL are robust, they should be interpreted with caution and validated in more diverse and longitudinal designs.

## Conclusions

Our results show that higher levels of general PF and its main components are closely associated with most dimensions of HRQoL among nursing students. Gender-specific patterns were observed: men showed better PF and higher psychological well-being and overall QoL perception. On the other hand, no statistically significant association was observed between PF and the social dimension in men, and flexibility showed more consistent benefits for HRQoL in women. The strongest associations were found between general PF and the different domains of HRQoL in both men and women.

Taken together, these findings underscore the need for future longitudinal studies incorporating objective fitness assessments to clarify the mechanisms underlying the observed associations. While the results may inform the development of tailored, gender-sensitive health promotion strategies, any intervention-related implications should be regarded as exploratory and require confirmation through longitudinal and interventional research.

Furthermore, the results suggest that comprehensive physical activity programs may be considered as a potential strategy for nursing students and other university populations, aimed at fostering the balanced development of all components of PF. Such programs may not only enhance HRQoL during university years but also contribute to the promotion of long-term physical and mental well-being.

## Supplementary Information

Supplementary Material 1.

Supplementary Material 2.

## Data Availability

The dataset supporting the findings of this study has been deposited in the open-access repository Zenodo (https://doi.org/10.5281/zenodo.14710329). However, access to the dataset is restricted due to ethical and privacy considerations. Data are available from the corresponding author upon reasonable request and with approval from the relevant institutional ethics committee.

## Abbreviations

HRQoL: Health-related Quality of Life
PF: Physical Fitness
WHOQOL-BREF: World Health Organization Quality of Life Brief
IFIS: International Fitness Scale
CRF: Cardiorespiratory Fitness
MS: Muscular Strength
QoL: Quality of Life
ANCOVA: analysis of covariance
BMI: Body Mass Index
WHO: World Health Organization
S-A: Speed-Agility
VIF: Variance Inflation Factor
PWB: Physical Well-being
LS: Life Satisfaction
PsWB: Psychological Well-being
