# Supplementary material for: Physical fitness and health-related quality of life in nursing students: a cross-sectional study with a gender perspective
Source: BMC Public Health. 2026 Mar 21;26:1404. doi: 10.1186/s12889-026-27062-4 (PMC13126776; doi:10.1186/s12889-026-27062-4)
Supplement: Supplementary file 1 — Supplementary Material 1. [file 12889_2026_27062_MOESM1_ESM.docx]

**Supplementary Table 1.** Mean differences (SE) in health-related quality of life by physical fitness categories, controlling for confounders by sex.

|  |  |  |  | **General PF - mean (SD)** | | | | | | |  | | |  | |
| --- | --- | --- | --- | --- | --- | --- | --- | --- | --- | --- | --- | --- | --- | --- | --- |
|  |  |  | **n** | **Very bad** | **n** | **Bad** | **n** | **Acceptable** | **n** | **Good** | **n** | **Very good** | ***p*** | |  |
| **PWB** | Total | | 9 | 60.44 (14.73) | 52 | 63.67 (16.82) | 256 | 68.24 (13.84) | 239 | 74.69 (12.12) | 54 | 76.32 (13.25) | **<0.001** | |  |
|  | Women | | 7 | 61.71 (16.76) | 42 | 64.18 (16.20) | 203 | 67.98 (14.18) | 179 | 74.21 (12.36) | 34 | 74.18 (14.45) | **<0.001** | |  |
|  | Men | | 2 | 56.00 (0) | 10 | 61.40 (20.15) | 53 | 69.44 (12.52) | 60 | 75.95 (11.62) | 20 | 78.95 (10.40) | **0.001** | |  |
| **PsWB** | Total | | 9 | 43.78 (15.01) | 51 | 56.36 (18.57) | 257 | 59.71 (15.66) | 240 | 67.14 (14.66) | 54 | 73.68 (13.49) | **<0.001** | |  |
|  | Women | | 7 | 42.86 (17.12) | 42 | 56.20 (18.71) | 203 | 59.50 (15.10) | 180 | 66.33 (14.83) | 34 | 71.44 (13.06) | **<0.001** | |  |
|  | Men | | 2 | 47.00 (4.24) | 9 | 57.11 (18.96) | 54 | 61.31 (17.01) | 60 | 70.17 (14.05) | 20 | 76.33 (13.50) | **<0.001** | |  |
| **Social** | Total | | 9 | 52.78 (22.34) | 52 | 67.33 (21.38) | 259 | 67.75 (18.50) | 236 | 72.87 (16.51) | 54 | 76.45 (16.81) | **<0.001** | |  |
|  | Women | | 7 | 51.71 (24.65) | 42 | 66.61 (22.23) | 204 | 67.13 (18.56) | 177 | 73.07 (17.22) | 34 | 77.38 (14.68) | **<0.001** | |  |
|  | Men | | 2 | 56.50 (17.68) | 10 | 70.50 (17.87) | 55 | 69.54 (18.50) | 59 | 72.32 (15.11) | 20 | 73.81 (19.62) | 0.483 | |  |
| **Environment** | Total | | 9 | 58.22 (16.23) | 52 | 65.63 (13.59) | 257 | 66.04 (14.03) | 240 | 71.87 (12.58) | 54 | 75.79 (12.44) | **<0.001** | |  |
|  | Women | | 7 | 62.43 (14.36) | 42 | 65.89 (14.18) | 202 | 65.77 (13.74) | 182 | 71.21 (13.08) | 34 | 75.06 (12.62) | **<0.001** | |  |
|  | Men | | 2 | 43.50 (17.68) | 10 | 64.50 (11.22) | 55 | 67.25 (15.34) | 58 | 73.48 (10.03) | 20 | 76.10 (12.04) | **<0.001** | |  |
| **LS** | Total | | 9 | 41.67 (17.68 | 54 | 58.80 (25.31) | 264 | 63.92 (21.20) | 249 | 71.49 (18.38) | 56 | 80.36 (18.90) | **<0.001** | |  |
|  | Women | | 7 | 46.43 (17.25) | 44 | 60.80 (24.35) | 206 | 63.96 (20.42) | 182 | 71.43 (19.16) | 34 | 79.41 (19.92) | **<0.001** | |  |
|  | Men | | 2 | 25.00 (0) | 10 | 50.00 (28.87) | 58 | 63.43 (25.14) | 67 | 71.67 (16.89) | 22 | 80.95 (17.51) | **<0.001** | |  |
| **Overall QoL** | Total | | 9 | 44.44 (16.67) | 54 | 56.02 (19.39) | 263 | 62.64 (18.39) | 249 | 70.98 (15.69) | 56 | 84.38 (15.49) | **<0.001** | |  |
|  | Women | | 7 | 46.43 (17.25) | 44 | 56.25 (19.53) | 205 | 62.20 (17.61) | 182 | 70.60 (16.03) | 34 | 81.62 (15.46) | **<0.001** | |  |
|  |  | Men | 2 | 37.50 (17.68) | 10 | 55.00 (19.72) | 58 | 64.35 (20.95) | 67 | 72.08 (15.33) | 22 | 88.10 (15.04) | **<0.001** | |  |

PF: physical fitness; QoL: Quality of Life; LS: Life satisfaction; PWB: Physical Well-being; PsWB: Psychological well-being.

**In bold:** statistically significant relationships.

**Table S1 (cont.).** Mean differences (SE) in health-related quality of life by physical fitness categories, controlling for confounders by sex.

|  | **Cardiorespiratory fitness - mean (SD)** | | | | | | | | | | | |  |
| --- | --- | --- | --- | --- | --- | --- | --- | --- | --- | --- | --- | --- | --- |
|  |  | **n** | **Very bad** | **n** | **Bad** | **n** | **Acceptable** | **n** | **Good** | **n** | **Very good** | ***p*** |  |
| **PWB** | Total | 15 | 65.59 (14.85) | 145 | 68.53 (14.58) | 235 | 69.73 (14.33) | 181 | 73.61 (12.64) | 43 | 77.16 (12,58) | **0.001** |  |
|  | Women | 14 | 66.19 (15.13) | 117 | 68.14 (15.02) | 176 | 69.41 (14.30) | 128 | 72.91 (12.92) | 29 | 76.24 (12,66) | **0.010** |  |
|  | Men | 1 | 56.00 (0) | 28 | 70.23 (12.36) | 59 | 70.30 (14.69) | 53 | 75.02 (11.80) | 14 | 79.07 (12,64) | 0.145 |  |
| **PsWB** | Total | 15 | 57.06 (18.03) | 147 | 59.29 (16.41) | 233 | 62.54 (15.65) | 182 | 65.40 (15.77) | 44 | 75.50 (13,41) | **<0.001** |  |
|  | Women | 14 | 57.50 (18.53) | 118 | 58.56 (16.32) | 175 | 62.46 (15.19) | 128 | 63.98 (15.49) | 30 | 73.67 (14,34) | **<0.001** |  |
|  | Men | 1 | 50.00 (0) | 29 | 62.89 (15.62) | 58 | 63.63 (17.03) | 54 | 68.38 (16.11) | 14 | 79.43 (10,58) | **0.010** |  |
| **Social** | Total | 15 | 62.53 (26.09) | 145 | 70.70 (18.29) | 235 | 69.17 (17.80) | 181 | 70.27 (17.83) | 44 | 77.55 (17,45) | **0.045** |  |
|  | Women | 14 | 63.69 (26.49) | 116 | 70.45 (19.14) | 175 | 68.07 (18.07) | 128 | 70.45 (18.02) | 30 | 78.53 (16,22) | **0.027** |  |
|  | Men | 1 | 44.00 (0) | 29 | 71.52 (14.63) | 60 | 72.33 (17.38) | 53 | 69.08 (17.44) | 14 | 75.43 (20,33) | 0.512 |  |
| **Environment** | Total | 15 | 67.76 (15.63) | 147 | 66.41 (13.21) | 235 | 67.55 (14.01) | 181 | 71.34 (13.01) | 43 | 77.07 (13,12) | **<0.001** |  |
|  | Women | 14 | 70.06 (12.84) | 118 | 65.84 (13.15) | 176 | 67.15 (14.56) | 128 | 70.83 (12.49) | 30 | 76.43 (13,60) | **0.004** |  |
|  | Men | 1 | 31.00 (0) | 29 | 68.67 (14.10) | 59 | 68.17 (11.19) | 53 | 72.43 (13.96) | 13 | 78.54 (12,32) | **0.003** |  |
| **LS** | Total | 17 | 58.82 (21.53) | 148 | 62.33 (21.53) | 238 | 66.39 (20.52) | 185 | 70.68 (19.89) | 44 | 82.49 (19,12) | **<0.001** |  |
|  | Women | 16 | 60.94 (25.77) | 119 | 63.03 (20.80) | 179 | 66.76 (19.97) | 129 | 69.96 (20.58) | 30 | 80.83 (20,43) | **0.001** |  |
|  | Men | 1 | 50.00 (0) | 29 | 57.69 (24.26) | 59 | 65.09 (23.17) | 56 | 71.70 (18.37) | 14 | 84.71 /16,16) | **0.002** |  |
| **Overall QoL** | Total | 15 | 63.33 (22.89) | 145 | 58.79 (18.76) | 236 | 65.89 (17.00) | 183 | 71.31 (17.48) | 44 | 84.09 (14,36) | **<0.001** |  |
|  | Women | 14 | 66.07 (21.05) | 117 | 58.97 (17.82) | 177 | 64.27 (16.79) | 129 | 71.32 (17.71) | 30 | 81.67 (14,58) | **<0.001** |  |
|  | Men | 1 | 25.00 (0) | 25 | 56.00 (21.98) | 53 | 71.23 (17.26) | 51 | 71.08 (16.86) | 14 | 89.29 (12,84) | **<0.001** |  |

PF: physical fitness; QoL: Quality of Life; LS: Life satisfaction; PWB: Physical Well-being; PsWB: Psychological well-being.

**In bold:** statistically significant relationships.

**Table S1(Cont.).** Mean differences (SE) in health-related quality of life by physical fitness categories, controlling for confounders by sex.

|  | | | **Muscular strength - mean (SD)** | | | | | | | | | |
| --- | --- | --- | --- | --- | --- | --- | --- | --- | --- | --- | --- | --- |
|  |  | **n** | **Very bad** | **n** | **Bad** | **n** | **Acceptable** | **n** | **Good** | **n** | **Very good** | ***p*** |
| **PWB** | Total | 13 | 62.15 (18.03) | 86 | 68.40 (13,67) | 245 | 68.99 (14.33) | 216 | 72.59 (12,96) | 59 | 79.17 (12.33) | **<0.001** |
|  | Women | 13 | 63.17 (18.44) | 75 | 68.01 (13,74) | 193 | 69.21 (14.37) | 153 | 72.25 (13,57) | 31 | 76.58 (13.20) | **0.004** |
|  | Men | 0 |  | 11 | 69.27 (13,02) | 52 | 67.87 (15.04) | 63 | 73.41 (11,39) | 28 | 81.12 (10.67) | **<0.001** |
| **PsWB** | Total | 13 | 52.92 (22.53) | 86 | 59.74 (18,21) | 245 | 60.40 (14.12) | 216 | 66.54 (16,33) | 61 | 71.44 (14.61) | **<0.001** |
|  | Women | 13 | 55.25 (21.84) | 75 | 59.79 (17,76) | 193 | 60.12 (14.02) | 152 | 65.94 (16,21) | 33 | 68.33 (15.25) | **<0.001** |
|  | Men | 0 |  | 11 | 57.45 (21,63) | 52 | 62.56 (14.84) | 64 | 67.97 (16,66) | 28 | 74.15 (13.10) | **0.005** |
| **Social** | Total | 13 | 66.31 (24.46) | 86 | 66.56 (19,71) | 245 | 68.86 (18.56) | 217 | 72.25 (17,02) | 59 | 75.08 (11.61) | **0.036** |
|  | Women | 13 | 67.17 (25.34) | 75 | 65.72 (19,89) | 192 | 68.61 (18.79) | 153 | 72.79 (17,37) | 31 | 73.97 (16.35) | **0.022** |
|  | Men | 0 |  | 11 | 69.82 (17,90) | 53 | 69.41 (19.05) | 64 | 70.95 (16,19) | 28 | 75.23 (16.54) | 0.527 |
| **Environment** | Total | 13 | 63.69 (13.45) | 86 | 66.09 (14,43) | 245 | 66.90 (13.93) | 217 | 70.84 (12,95) | 60 | 76.78 (11.61) | **<0.001** |
|  | Women | 13 | 63.75 (14.05) | 75 | 66.05 (14,10) | 192 | 67.25 (14.02) | 154 | 70.06 (12,99) | 33 | 75.91 (12.40) | 0.007 |
|  | Men | 0 |  | 9 | 65.55 (17,65) | 53 | 65.40 (13.52) | 63 | 72.73 (12,77) | 27 | 76.32 (9.51) | **0.001** |
| **LS** | Total | 13 | 63.46 (24.19) | 88 | 61.93 (22,74) | 250 | 64.70 (20.93) | 220 | 70.68 (19,95) | 61 | 77.46 (19.74) | **<0.001** |
|  | Women | 12 | 64.58 (24.91) | 76 | 61.51 (20,99) | 196 | 65.43 (20.44) | 156 | 71.31 (20,44) | 33 | 75.00 (20.73) | **0.004** |
|  | Men | 1 | 50.00 (0) | 12 | 61.36 (32,33) | 46 | 60.87 (23.95) | 64 | 69.15 (18,76) | 26 | 79.81 (18.73) | **<0.001** |
| **Overall QoL** | Total | 12 | 60.41 (19.82) | 86 | 58.72 (18,70) | 247 | 64.57 (18.32) | 217 | 69.93 (17,27) | 61 | 79.92 (16.34) | **<0.001** |
|  | Women | 12 | 61.36 (20.50) | 75 | 57.33 (17,32) | 194 | 64.69 (17.72) | 154 | 70.13 (17,64) | 33 | 76.51 (16.46) | **<0.001** |
|  | Men | 0 |  | 11 | 65.00 (24,15) | 53 | 63.89 (21.69) | 63 | 69.44 (16,46) | 28 | 83.65 (15.72) | **0.001** |

QoL: Quality of Life; LS: Life satisfaction; PWB: Physical Well-being; PsWB: Psychological well-being.

**In bold:** statistically significant relationships.

**Table S1(Cont.).** Mean differences (SE) in health-related quality of life by physical fitness categories, controlling for confounders by sex.

|  | | **Velocity-agility - mean (SD)** | | | | | | | | | | | |
| --- | --- | --- | --- | --- | --- | --- | --- | --- | --- | --- | --- | --- | --- |
|  |  | **n** | **Very bad** | **n** | **Bad** | **n** | **Acceptable** | **n** | **Good** | **n** | **Very good** | ***p*** |  |
| **PWB** | Total | 12 | 60.23 (18.99) | 75 | 65.52 (16.14) | 240 | 68.80 (13.14) | 243 | 74.35 (12.88) | 49 | 76.02 (13.11) | **<0.001** |  |
|  | Women | 11 | 64.91 (15.10) | 61 | 64.54 (16.52) | 191 | 68.62 (13.44) | 175 | 74.05 (13.09) | 27 | 73.93 (14.37) | **<0.001** |  |
|  | Men | 1 | 19.00 (0) | 14 | 69.78 (14.15) | 49 | 69.38 (12.26) | 68 | 74.64 (12.23) | 22 | 78.60 (11.32) | **<0.001** |  |
| **PsWB** | Total | 11 | 49.00 (20.59) | 74 | 58.24 (17.06) | 242 | 60.64 (14.58) | 244 | 66.81 (15.24) | 50 | 70.62 (19.78) | **<0.001** |  |
|  | Women | 11 | 51.18 (20.09) | 60 | 56.93 (17.11) | 192 | 60.48 (14.47) | 175 | 66.18 (15.24) | 28 | 68.39 (19.13) | **<0.001** |  |
|  | Men | 0 |  | 14 | 63.86 (16.24) | 50 | 62.02 (14.76) | 69 | 67.99 (15.25) | 22 | 74.55 (21.31) | **0.033** |  |
| **Social** | Total | 12 | 58.69 (21.12) | 74 | 68.24 (19.32) | 241 | 68.34 (17.72) | 242 | 72.37 (17.90) | 51 | 75.10 (18.23) | **0.009** |  |
|  | Women | 11 | 57.45 (22.50) | 60 | 68.67 (20.05) | 190 | 67.58 (17.94) | 174 | 72.77 (17.87) | 29 | 74.14 (20.66) | **0.009** |  |
|  | Men | 1 | 75.00 (0) | 14 | 66.43 (16.33) | 51 | 70.60 (17.22) | 68 | 70.65 (18.15) | 22 | 77.15 (14.78) | 0.551 |  |
| **Environment** | Total | 12 | 65.46 (15.36) | 75 | 64.47 (14.26) | 241 | 65.95 (13.27) | 242 | 72.16 (13.21) | 51 | 76.35 (11.37) | **<0.001** |  |
|  | Women | 11 | 66.55 (16.50) | 61 | 63.84 (14.04) | 190 | 65.69 (13.19) | 176 | 71.79 (13.05) | 29 | 77.52 (12.27) | **<0.001** |  |
|  | Men | 1 | 56.00 (0) | 14 | 67.21 (15.40) | 51 | 67.38 (13.97) | 66 | 72.66 (13.15) | 22 | 73.55 (10.72) | 0.290 |  |
| **LS** | Total | 13 | 51.92 (31.39) | 75 | 62.67 (21.11) | 244 | 65.06 (20.69) | 250 | 70.80 (19.97) | 50 | 75.50 (22.30) | **0.005** |  |
|  | Women | 12 | 56.82 (29.77) | 61 | 62.70 (20.22) | 194 | 65.72 (20.14) | 179 | 70.11 (20.54) | 28 | 75.89 (23.06) | **0.044** |  |
|  | Men | 1 | 0 (0) | 14 | 62.50 (25.48) | 50 | 61.67 (22.99) | 71 | 72.01 (18.73) | 22 | 75.00 (22.94) | **0.024** |  |
| **Overall QoL** | Total | 11 | 63.64 (17.19) | 75 | 60.33 (19.32) | 236 | 62.71 (17.79) | 243 | 70.68 (17.51) | 48 | 79.69 (10.06) | **<0.001** |  |
|  | Women | 10 | 65.00 (17.48) | 61 | 59.43 (17.77) | 191 | 62.43 (17.93) | 177 | 70.06 (17.28) | 28 | 80.36 (15.75) | **<0.001** |  |
|  | Men | 1 | 50.00(0) | 14 | 64.29 (25.41) | 45 | 64.77 (16.45) | 66 | 72.31 (18.29) | 20 | 78.75 (19.74) | 0.249 |  |

QoL: Quality of Life; LS: Life satisfaction; PWB: Physical Well-being; PsWB: Psychological well-being.

**In bold:** statistically significant relationships.

**Table S1 (Cont.).** Mean differences (SE) in health-related quality of life by physical fitness categories, controlling for confounders by sex.

|  | | **Flexibilidad - mean (SD)** | | | | | | | | | | |
| --- | --- | --- | --- | --- | --- | --- | --- | --- | --- | --- | --- | --- |
|  |  | **n** | **Muy mala** | **n** | **Mala** | **n** | **Aceptable** | **n** | **Buena** | **n** | **Muy buena** | ***p*** |
| **PWB** | Total | 38 | 70,18 (15,17) | 135 | 69,86 (13,85) | 210 | 70,30 (13,86) | 156 | 71,53 (14,01) | 69 | 74,46 (14,30) | 0,302 |
|  | Women | 21 | 70,38 (14,25) | 108 | 68,29 (13,79) | 154 | 69,24 (14,21) | 127 | 71,67 (13,97) | 53 | 74,72 (15,11) | 0,113 |
|  | Men | 17 | 70,35 (17,04) | 27 | 75,36 (12,63) | 56 | 73,05 (12,26) | 29 | 70,37 (14,44) | 16 | 73,94 (12,13) | 0,632 |
| **PsWB** | Total | 38 | 61,47 (17, 38) | 135 | 62,26 (16,26) | 214 | 63,34 (15,66) | 161 | 64,51 (15,45) | 71 | 64,24 (19,39) | 0,832 |
|  | Women | 21 | 59,67 (19,37) | 107 | 59,69 (16,25) | 154 | 62,29 (15,22) | 129 | 64,83 (14,96) | 53 | 64,06 (18,23) | 0,212 |
|  | Men | 16 | 64,56 (14,87) | 27 | 72,04 (12,55) | 56 | 66,54 (15,34) | 29 | 62,97 (18,19) | 17 | 66,35 (22,72) | 0,249 |
| **Social** | Total | 38 | 70,34 (19,71) | 135 | 67,57 (18,67) | 214 | 71,31 (18,23) | 161 | 69,88 (15,54) | 70 | 73,50 (20,24) | 0,366 |
|  | Women | 20 | 69,35 (23,97) | 107 | 67,10 (19,06) | 154 | 70,79 (18,45) | 129 | 70,32 (16,85) | 52 | 72,25 (20,62) | 0,489 |
|  | Men | 17 | 70,88 (14,42) | 27 | 68,93 (17,48) | 56 | 71,98 (17,71) | 29 | 68,13 (16,13) | 17 | 77,59 (19,70) | 0,497 |
| **Environment** | Total | 39 | 67,36 (13,76) | 137 | 68,88 (13,35) | 212 | 69,01 (13,87) | 160 | 67,98 (13,58) | 71 | 73,31 (14,33) | 0,126 |
|  | Women | 21 | 68,10 (13,99) | 108 | 67,75 (13,20 | 153 | 68,61 (14,12) | 130 | 67,52 (12,90) | 53 | 73,04 (15,37) | 0,298 |
|  | Men | 17 | 66,71 (14,24) | 28 | 72,31 (12,90) | 55 | 70,25 (13,01) | 28 | 68,45 (15,81) | 17 | 75,18 (10,41 | 0,419 |
| **LS** | Total | 39 | 63,46 (26,19) | 139 | 64,57 (21,05) | 218 | 68,69 (20,02) | 164 | 67,84 (20,86) | 70 | 71,79 (22,89) | 0,276 |
|  | Women | 21 | 67,86 (26,39) | 110 | 62,73 (21,35) | 157 | 68,47 (19,64) | 130 | 67,69 (20,28) | 53 | 72,64 (22,61) | 0,126 |
|  | Men | 18 | 57,35 (26,17) | 29 | 71,43 (18,90) | 61 | 68,75 (20,92) | 34 | 67,50 (24,70) | 17 | 70,31 (24,53) | 0,331 |
| **Overall QoL** | Total | 38 | 59,87 (17,95) | 135 | 63,15 (19,75) | 211 | 67,53 (17,94) | 158 | 67,72 (17,46) | 69 | 74,28 (20,10) | **0,001** |
|  | Women | 21 | 60,71 (14,94) | 108 | 60,42 (18,76) | 155 | 66,77 (17,82) | 128 | 67,77 (16,94) | 53 | 73,11 (20,13) | **<0,001** |
|  | Men | 17 | 58,82 (21,54) | 27 | 74,07 (20,19) | 55 | 70,45 (17,41) | 29 | 67,24 (20,16) | 16 | 78,12 (20,16) | 0,062 |

QoL: Quality of Life; LS: Life satisfaction; PWB: Physical Well-being; PsWB: Psychological well-being. **In bold:** statistically significant relationships.
