# Supplementary material for: Physical fitness and health-related quality of life in nursing students: a cross-sectional study with a gender perspective
Source: BMC Public Health. 2026 Mar 21;26:1404. doi: 10.1186/s12889-026-27062-4 (PMC13126776; doi:10.1186/s12889-026-27062-4)
Supplement: Supplementary file 2 — Supplementary Material 2. [file 12889_2026_27062_MOESM2_ESM.docx]

**Supplementary Table 2.** Independent association between cardiorespiratory fitness, muscle strength, speed-agility and flexibility, with health-related quality of life, controlling body mass index and parents' educational level, by gender.

|  | **Total** |  |  | **Women** |  |  | **Men** |  |  |
| --- | --- | --- | --- | --- | --- | --- | --- | --- | --- |
|  | **R^2^** | **ß** | **p** | **R^2^** | **ß** | **p** | **R^2^** | **ß** | **p** |
| **Overall QoL** |  |  |  |  |  |  |  |  |  |
| **General PF** | 0.179 | 0.381 | **<0.001** | 0.154 | 0.345 | **<0.001** | 0.283 | 0.430 | **<0.001** |
| **CRF** | 0.133 | 0.314 | **<0.001** | 0.110 | 0.273 | **<0.001** | 0.243 | 0.373 | **<0.001** |
| **MS** | 0.122 | 0.294 | **<0.001** | 0.105 | 0.161 | **<0.001** | 0.236 | 0.365 | **<0.001** |
| **V-A** | 0.092 | 0.239 | **<0.001** | 0.089 | 0.231 | **<0.001** | 0.136 | 0.176 | **0.035** |
| **Flex** | 0.064 | 0.161 | **<0.001** | 0.075 | 0.190 | **<0.001** | 0.117 | 0.093 | 0.251 |
| **LS** |  |  |  |  |  |  |  |  |  |
| **General PF** | 0.106 | 0.288 | **<0.001** | 0.075 | 0.248 | **<0.001** | 0.214 | 0.382 | **<0.001** |
| **CRF** | 0.072 | 0.220 | **<0.001** | 0.054 | 0.178 | **<0.001** | 0.174 | 0.318 | **<0.001** |
| **MS** | 0.067 | 0.207 | **<0.001** | 0.052 | 0.171 | **<0.001** | 0.192 | 0.349 | **<0.001** |
| **V-A** | 0.052 | 0.168 | **<0.001** | 0.043 | 0.141 | **0.003** | 0.116 | 0.211 | **0.012** |
| **Flex** | 0.032 | 0.080 | **0.047** | 0.033 | 0.091 | 0.049 | 0.077 | 0.038 | 0.645 |
| **PWB** |  |  |  |  |  |  |  |  |  |
| **General PF** | 0.091 | 0.266 | **<0.001** | 0.073 | 0.231 | **<0.001** | 0.157 | 0.341 | **<0.001** |
| **CRF** | 0.054 | 0.183 | **<0.001** | 0.046 | 0.162 | **<0.001** | 0.087 | 0.202 | **0.015** |
| **MS** | 0.070 | 0.223 | **<0.001** | 0.052 | 0.177 | **<0.001** | 0.151 | 0.329 | **<0.001** |
| **V-A** | 0.076 | 0.240 | **<0.001** | 0.064 | 0.212 | **<0.001** | 0.118 | 0.280 | **0.001** |
| **Flex** | 0.027 | 0.072 | 0.076 | 0.033 | 0.108 | **0.020** | 0.048 | -0.023 | 0.785 |
| **PsWB** |  |  |  |  |  |  |  |  |  |
| **General PF** | 0.111 | 0.324 | **<0.001** | 0.095 | 0.289 | **<0.001** | 0.155 | 0.390 | **<0.001** |
| **CRF** | 0.061 | 0.231 | **<0.001** | 0.053 | 0.200 | **<0.001** | 0.084 | 0.277 | **<0.001** |
| **MS** | 0.068 | 0.245 | **<0.001** | 0.055 | 0.202 | **<0.001** | 0.098 | 0.304 | **<0.001** |
| **V-A** | 0.066 | 0.244 | **<0.001** | 0.064 | 0.228 | **<0.001** | 0.059 | 0.230 | **0.008** |
| **Flex** | 0.012 | 0.047 | 0.247 | 0.025 | 0.101 | **0.030** | 0.016 | -0.077 | 0.365 |
| **Social** |  |  |  |  |  |  |  |  |  |
| **General PF** | 0.043 | 0.185 | **<0.001** | 0.049 | 0.205 | **<0.001** | 0.038 | 0.120 | 0.159 |
| **CRF** | 0.016 | 0.077 | 0.061 | 0.017 | 0.090 | 0.060 | 0.025 | 0.017 | 0.837 |
| **MS** | 0.030 | 0.145 | **<0.001** | 0.031 | 0.153 | **0.001** | 0.034 | 0.102 | 0.231 |
| **V-A** | 0.029 | 0.142 | **<0.001** | 0.029 | 0.148 | **0.002** | 0.035 | 0.107 | 0.220 |
| **Flex** | 0.013 | 0.055 | 0.175 | 0.013 | 0.061 | 0.189 | 0.026 | 0.043 | 0.610 |
| **Environment** |  |  |  |  |  |  |  |  |  |
| **General PF** | 0.111 | 0.225 | **<0.001** | 0.990 | 0.180 | **<0.001** | 0.166 | 0.328 | **<0.001** |
| **CRF** | 0.088 | 0.162 | **<0.001** | 0.084 | 0.128 | **0.005** | 0.115 | 0.229 | **0.005** |
| **MS** | 0.101 | 0.198 | **<0.001** | 0.091 | 0.155 | **<0.001** | 0.154 | 0.307 | **<0.001** |
| **V-A** | 0.105 | 0.213 | **<0.001** | 0.111 | 0.213 | **<0.001** | 0.089 | 0.165 | 0.054 |
| **Flex** | 0.065 | 0.044 | 0.267 | 0.071 | 0.047 | 0.295 | 0.067 | 0.052 | 0.528 |

QoL: Quality of Life; LS: Life satisfaction; PWB: Physical Well-being; PsWB: Psychological well-being; PF: physical fitness; CRF: Cardiorespiratory fitness; MS: Muscular strength; V-A: Velocity-Agility; Flex: Flexibility. **In bold:** statistically significant relationships.
